# Interleukin-6 levels and their haplotypes are associated with serological autoantibodies status and clinical activity in rheumatoid arthritis

**DOI:** 10.3389/fimmu.2025.1679644

**Published:** 2025-11-06

**Authors:** Ma. Natividad Flores-Castro, Ramcés Falfán-Valencia, Olivia Briceño, Ilse Adriana Gutiérrez-Pérez, Oscar Zaragoza-García, Gloria Pérez-Rubio, Cristina Morales-Martínez, Iris Paola Guzmán-Guzmán

**Affiliations:** 1 Laboratory of Multidisciplinary Research and Biomedical Innovation, Faculty of Chemical-Biological Sciences, Autonomous University of Guerrero, Chilpancingo, Mexico; 2 Pneumogenomics Laboratory, Instituto Nacional de Enfermedades Respiratorias Ismael Cosío Villegas, Mexico City, Mexico; 3 Infectious Diseases Research Center, Instituto Nacional de Enfermedades Respiratorias Ismael Cosío Villegas, Mexico City, Mexico; 4 Hospital IMSS-Bienestar Dr. José G. Parres, Cuernavaca, Mexico

**Keywords:** IL-6, haplotype, clinical activity, serological status, rheumatoid arthritis

## Abstract

**Introduction:**

Interleukin-6 (IL-6) plays an important role in the pathogenesis, progression, and severity of rheumatoid arthritis (RA). High levels of IL-6 are involved in the maintenance of inflammation in the synovium and the development of new blood vessels in the inflamed synovium, contributing to pannus formation. In the present study, we aimed to investigate the relationship of IL-6 polymorphisms/ haplotypes with RA in Mexican patients and on circulating levels of IL-6 and the clinical features of the disease.

**Methods:**

A total of 225 patients with RA and 362 healthy controls were recruited. Patients’ clinical features were collected, and inflammatory and serological parameters [anti-cyclic citrullinated peptides (anti-CCPs) and rheumatoid factor (RF)] were assessed. All subjects were genotyped for IL-6 polymorphisms (rs1800797, rs1800796, and rs1818879) using real-time polymerase chain reaction (PCR) with TaqMan probes. Circulating levels of IL-6 were measured by commercial ELISA (enzyme-linked immunosorbent assay) kits.

**Results and discussion:**

Our results show that the GGG haplotype of the IL-6 gene is associated with a serological status of double-positive autoantibodies [odds ratio (OR) = 2.09, p = 0.009], as well as with a double-positive high autoantibodies status (OR = 2.08, p = 0.04), although a marginal association was shown to RA susceptibility (OR = 1.34, p = 0.050). Polymorphisms and haplotypes were not associated with serum levels of IL-6. However, serum levels of IL-6 ≥27.6 pg/mL were associated with clinical features of RA severity, such as morning stiffness ≥30 min (OR = 1.83, p = 0.001), HAQ-DI ≥1 (OR = 2.73, p=0.009), DAS28-ESR ≥3.2 (OR = 3.63, p = 0.002), and hsCRP ≥10 mg/L (OR = 6.35, p < 0.001). This study provides evidence of the relationship between IL-6 haplotypes, circulating levels of IL-6, and clinical features and serological status to autoantibodies in patients with RA from Mexico.

## 1 Introduction

Rheumatoid arthritis (RA) is a systemic autoimmune disease characterized by inflammation of synovial joints, cartilage loss, and subcondral bone erosions. During RA, the persistence of the inflammatory process, the uncontrolled immune response, synovial proliferation, disease progression, and damage to both cartilage and bone are orchestrated by proinflammatory cytokines, including interleukin-6 (IL-6) (1–3).

Some cells, such as T lymphocytes, B lymphocytes, monocytes, fibroblasts, endothelial cells, and synoviocytes, can produce and secrete IL-6 (4), and binding with its receptor (IL-6/IL-6R) activates the JAK-STAT pathway. When IL-6 binds to the soluble receptor (IL-6/sIL-6R), cell recruitment is promoted, whereas when IL-6 binds to the membrane receptor (IL-6/IL-6R), systemic inflammation is promoted by increasing the synthesis of acute phase proteins such as C-reactive protein, serum amyloid A, fibrinogen, alpha-1-antitrypsin, alpha-1-antichymotrypsin, and haptoglobin (2, 4, 5). IL-6 is also known to be a cytokine required for immunoglobulin class switching and plays an important role in acquired immune response by stimulating effector T-cell development (5–7).

Higher serum IL-6 levels have been found in patients with RA with high clinical activity and higher radiological grades and in those with functional disability (8, 9). Moreover, it has been shown that IL-6 expression might be, in part, genetically modulated by single-nucleotide polymorphisms (SNPs) in RA (1, 10). Several SNPs have been described in the *IL-6* gene, which are mainly located on regulatory regions, which have been associated with the transcription of the gene with possible effects on the severity and susceptibility to develop RA (11, 12). Several SNPs have been reported on the promoter region of IL-6 located at position −597G/C (rs1800797) (13), −572G/C (rs1800796) (13–16), and −174G/C (rs1800795) (13, 15, 17, 18) in the promoter region of IL-6 and have been associated with susceptibility to RA. Interestingly, another SNP, rs1818879, located in the 3′ untranslated region (UTR), has been associated with chronic inflammatory and autoimmune diseases, such as multiple sclerosis (19), but it has not been studied in RA.

Recent research focused on *IL-6* SNPs has shown that specific haplotypes are associated with susceptibility to RA. On a Turkish cohort of patients with RA, the GG haplotype (rs1800795 and rs2069837) (20) and the haplotype GCCGCT (rs1800797, rs1800796, rs1800795, rs1524107, rs2069840, and rs1474347) in a Chinese RA cohort have been reported to be associated with RA susceptibility (13), but not another clinical characteristic in RA; however, these susceptibility results differed between populations. The present study analyzed the role of *IL-6* SNPs rs1800797, rs1800796, and rs1818879 and haplotypes with the susceptibility to RA, as well as the relationship of circulating levels of IL-6 with serological and inflammatory markers in a Mexican population with RA.

## 2 Materials and methods

### 2.1 Study population

A case–control study was conducted among patients with RA (*n* = 225, including both women and men). The diagnosis of patients with RA was based on the ACR/EULAR criteria 2010 (21). Participants were recruited from December 2017 to December 2020 at the Department of Internal Medicine from Hospital IMSS-Bienestar Dr. Raymundo Abarca Alarcón in Guerrero, Mexico. A general population group of 362 participants was considered, consisting of healthy controls from samples taken from the open population with no history of RA, autoimmune, musculoskeletal, or infectious diseases to determine the association with RA. The study was approved by the Research Committee Ethics of the Autonomous University of Guerrero, Mexico (approval code CB-004/2017). All participants signed a written informed consent.

### 2.2 Sociodemographic and clinical data

Demographic, clinical information, and antirheumatic treatment data were gathered from all patients during consultations and from their medical records. Clinical characteristics, such as morning stiffness, and tender and swollen joint count, were assessed. Furthermore, functional disability was evaluated using HAQ-DI (Health Assessment Questionnaire); a HAQ-DI score ≥1 denoted disability presence.

The Disease Activity Score in 28 joints with erythrocyte sedimentation rate (DAS28-ESR) were assessed. The scores for DAS28-ESR were categorized as follows: >5.1, ≥3.2 to ≤5.1, ≥2.6 to <3.2, and <2.6, representing high, moderate, low disease activity, and remission, respectively. Also, the joint damage was assessed using radiographs from hands, classifying the Sharp–van der Heijde (SvH) score into five categories: 0 = normal; 1 = asymmetrical or minimal narrowing up to a maximum of 25%; 2 = definite narrowing with loss of up to 50% of the normal space; 3 = definite narrowing with loss of 50%–99% of the normal space or subluxation; and, 4 = absence of joint space, presumptive evidence of ankylosis, or complete luxation.

The standard treatments were mainly methotrexate, received orally, in combination with chloroquine and corticosteroids (Cs) such as prednisone and non-steroidal anti-inflammatory drugs (NSAIDs); this study does not include patients treated with biological agents.

### 2.3 Serological status and inflammatory markers

Blood samples were collected after 8 h of overnight fasting via venipuncture and transferred to tubes with and without a clot activator. An erythrocyte sediment rate (ESR) test was performed using the Wintrobe method. The quantification of high-sensitivity C-reactive protein (hsCRP) and rheumatoid factor (RF) was evaluated by the immunoturbidimetry method, according to the manufacturer’s instructions (COBAS C311, Roche Diagnostics GmbH, Germany). IgG isotype antibodies against citrullinated peptides (anti-CCPs) were determined using an ELISA (enzyme-linked immunosorbent assay) commercial kit (DIASTAT anti-CCP Axis-Shield, Dundee, United Kingdom) following the manufacturer’s specifications and the Multiskan FC Microplaque Photometer automated plate reader (Thermo Scientific, Shanghai). Elevated levels of hsCRP were considered ≥10 mg/L; RF positive when >20 IU/mL; anti-CCPs positive when >5 U/mL; RF high when >60 IU/L; anti-CCPs high when >30 U/mL; double-positive autoantibodies when RF levels exceeded 20 IU/mL and anti-CCPs exceeded 5 U/mL; and double-positive high autoantibodies when RF levels exceeded 60 IU/mL and anti-CCPs exceeded 30 U/mL.

### 2.4 DNA extraction and TaqMan genotyping

Genomic DNA was extracted from peripheral blood, and SNPs were genotyped by allele discrimination using commercial TaqMan probes (Applied Biosystems, San Francisco, CA, USA). The analyzed SNPs included rs1800797/promoter, −597G/A (C_1839695_20), rs1800796/promoter, −572G/C (C_11326893_10), and rs1818879/3′UTR G/A (C_26518729_10), all of which are listed under the catalog number 4351379. These were evaluated with quantitative polymerase chain reaction (qPCR) on a 7300 Real-Time PCR System (Applied Biosystems/Thermo Fisher Scientific Inc., Singapore), following the manufacturer’s instructions. Amplifications were conducted under the following cycling conditions: one cycle at 60°C for 30 s, 95°C for 10 min; 40 cycles at 95°C for 15 s and 60°C for 1 min; and, finally, 60°C for 30 s. Genotype analysis was conducted using the sequence detection software in StepOne™ version 2.3 software (Applied Biosystems, CA, USA).

### 2.5 Determination of circulating levels of IL-6

Circulating levels of IL-6 were measured using a specific sandwich ELISA method (BioLegend Max™, Inc. San Diego, CA, USA, cat. 430507), following the manufacturer’s instructions on an automatized equipment Multiskan Go (Thermo Scientific, Finland). The absorbance was read at 450 nm using a standard curve, and IL-6 levels were quantified. The detection sensitivity for IL-6 was 1.6 pg/mL. In this study, IL-6 values were categorized as follows: low (<10.6 pg/mL), middle (10.6 to 27.29 pg/mL), and high (≥27.3 pg/mL), based on cutoff tertiles from the data of our cohort.

### 2.6 Statistical analysis

The categorical variables were expressed as proportions and compared using the chi-squared test. Nonparametric quantitative variables were presented with medians and 5th–95th percentiles. *IL-6* SNP-allele frequencies were calculated, and the Hardy–Weinberg equilibrium (HWE) exact test (estimation of *P* value) was performed for each group of participants (cases and controls) using STATA version 16 software. Differences in genotypes and allele frequencies between populations were measured using the chi-square test.

Genotype, allele, and haplotype frequencies for *IL-6* SNPs (rs1800797, rs1800796, and rs1818879) were estimated using the SHEsis online software (22). To analyze linkage disequilibrium (LD) between SNPs, we calculated Lewontin’s *D′* value using the SHEsis program, as well as the association between the haplotypes, RA susceptibility, clinical, and serological parameters.

The association between high levels of IL-6 with clinical and serological parameters was assessed with a logistic regression model adjusted for age, sex, and therapy scheme combined, providing odds ratios (ORs) and 95% confidence intervals (95% CI). Data analysis was performed using STATA version 16 (StataCorp, College Station, TX, USA) and GraphPad Prism v.8.4 (GraphPad Software, San Diego, CA, USA). In this study, values of *p* < 0.05 were considered statistically significant.

## 3 Results

### 3.1 Participant characteristics

The median age of the participants was 46 years for the RA group and 50 years for the control group. Ninety-two percent of our cohort was composed of women on the RA arm and 94% on the control group (**Table 1**). When analyzing the patients with RA, the median time since RA diagnosis was 6 years, with a morning stiffness median of 10 min, an HAQ-DI score of 0.21, a DAS28-ESR of 3.12, and an SvH score of 2. The levels of RF, anti-CCPs, and ESR were high on average. The median levels of hsCRP were 6.34 mg/L, and the median circulating levels of IL-6 were 15.90 pg/mL. All patients present an antirheumatic scheme with disease-modifying antirheumatic drugs (DMARDs) in monotherapy or combination (**Table 1**).

**Table 1:** Demographic, clinical, and treatment characteristics in patients with RA.

Characteristics	RA (*n* = 225)	Control subjects (*n* = 362)
Demographic data		
Age, years, median (P_5_–P_95_)^a^	46 (24–70.0)	50 (26–71)*
Sex, *n* (%)^b^		
Women	207 (92.0)	343 (94.75)
Men	18 (8.0)	19 (5.25)
Tobacco smoking, *n* (%)^b^	25 (11.11)	20 (5.52)*
Clinical assessment		
Duration of illness, years, median (P_5_–P_95_)^a^	6.0 (1–24)	–
Morning stiffness, min, median (P_5_–P_95_)^a^	10 (0–180)	–
HAQ-DI, score, median (P_5_–P_95_)^a^	0.21 (0–1.5)	–
VAS, score, median (P_5_–P_95_)^a^	30 (0–90)	–
DAS28-ESR, score, median (P_5_–P_95_)^a^	3.12 (1.93–6.72)	–
SvH, score, median (P_5_–P_95_)^a^	2 (1–4)	–
Serological and inflammatory markers		
Rheumatoid factor, IU/mL, median (P_5_–P_95_)^a^	140 (9.0–660.1)	
Anti-CCPs, U/mL, median (P_5_–P_95_)^a^	55.8 (0.4–431.55)	
ESR, mm/h, median (P_5_–P_95_)^a^	32 (10–55)	
hsCRP, mg/L, median (P_5_–P_95_)^a^	6.24 (0–53.45)	
IL-6, pg/mL, median (P_5_–P_95_)^a^	15.90 (4.53–157.74)	
Current therapy scheme		
Monotherapy DMARDs, *n* (%)^b^	104 (46.2)	–
Bitherapy or more DMARDs, *n* (%)^b^	121 (53.8)	–
Therapy scheme combined	152 (47.50)	–
DMARDs with or without NSAIDs, *n* (%)^b^	71 (31.56)	–
DMARDs with Cs *n* (%)^b^	154 (68.44)	–

Anti-CCPs, anti-cyclic citrullinated peptide antibodies; Cs, corticosteroids; DAS28, disease activity score 28; DMARDs, disease-modifying antirheumatic drugs; ESR, erythrocyte sedimentation rate; HAQ-DI, health assessment questionnaire disability index; hsCRP, high-sensitivity C-reactive protein; NSAIDs, nonsteroidal anti-inflamatory drugs; SvH, Sharp van der Heijde Score; VAS, Visual Analog Scale.

a Data are expressed as the median using percentiles 5th–95th and Mann–Whitney U test.

b Data are expressed as the n (%) using chi-square test. *p-value < 0.05.

### 3.2 *IL-6* polymorphisms and haplotype and their contribution to RA susceptibility

The distribution of genotypic and allelic frequencies of the three investigated SNPs is presented in **Table 2**. The genotypes and alleles of the SNPs were not associated with an increased risk of developing the disease in our cohort. However, the GG haplotype (rs1800797/rs1818879) was associated with the susceptibility to RA (OR = 1.34, 95% CI, 1.01–1.79, *p* = 0.03), while the GGG haplotype showed a trend toward RA susceptibility without reaching significance (OR = 1.34, 95% CI, 0.99–1.80, *p* = 0.05) (**Table 3**).

**Table 2:** Genotype and allele frequencies of polymorphisms in *IL-6* gene.

SNPs	RA (*n* = 225)	Control subjects (*n* = 362)	OR (95% CI), *p-*value
rs1800797			
GG, *n* (%)	212 (94.22)	327 (90.33)	1.0*
GA, *n* (%)	11 (4.89)	32 (8.84)	0.53 (0.23–1.10), 0.07
AA, *n* (%)	2 (0.89)	3 (0.83)	1.02 (0.08–9.05), 0.97
Dominant model			
GA+AA	13 (5.78)	35 (9.67)	1.74 (0.87–3.67), 0.09
Allele			
G, *n* (%)	435 (96.67)	686 (94.75)	1.0*
A, *n* (%)	15 (3.33)	38 (5.25)	0.62 (0.31–1.17), 0.12
HWE χ^2^, *p-*value	χ^2^ = 13.10 *p <* 0.001	χ^2^ = 4.48, *p* = 0.034	
rs1800796			
GG, *n* (%)	82 (36.44)	111 (30.66)	1.0*
GC, *n* (%)	104 (46.22)	178 (49.17)	0.79 (0.53–1.17), 0.21
CC, *n* (%)	39 (17.33)	73 (20.17)	0.72 (0.43–1.10), 0.18
Dominant model			
GC+CC	143 (63.55)	251 (69.34)	1.29 (0.89–1.87), 0.14
Allele			
G, *n* (%)	268 (59.56)	400 (55.25)	1.0*
C, *n* (%)	182 (40.44)	324 (44.75)	0.83 (0.65–1.07), 0.14
HWE χ^2^, *p-*value	χ^2^ = 0.36, *p* = 0.54	χ^2^ = 0.01, *p* = 0.91	
rs1818879			
GG, *n* (%)	20 (8.89)	19 (5.25)	1.0*
GA, *n* (%)	81 (36.0)	134 (37.02)	0.57 (0.27–1.21), 0.11
AA, *n* (%)	124 (55.11)	209 (57.73)	0.56 (0.27–1.16), 0.08
Dominant model			
GA+AA	205 (91.11)	343 (94.75)	1.76 (0.86–3.57), 0.08
Allele			
G, *n* (%)	121 (26.89)	172 (23.76)	1.0*
A, *n* (%)	329 (73.11)	552 (76.24)	0.84 (0.64–1.12), 0.22
HWE χ^2^, *p-*value	χ^2^ = 1.60, *p* = 0.20	χ^2^ = 0.17, *p* = 0.67	

CI, confidence interval; HWE, Hardy–Weinberg equilibrium; ND, not determined; OR, odds ratio; SNPs, single-nucleotide polymorphisms. The SNPs are listed in the order: IL-6 rs1800797_G>A, rs1800796_G>C, and rs1818879_G>A.

The OR and 95% CI, and *p* values were obtained by SHESIS test for haplotypes.

*p*-value <0.05 was considered statistically significant. 1.0* Reference category.

**Table 3:** Haplotype frequencies of polymorphisms in *IL-6* gene.

Haplotypes	RA (*n* = 225)	Control subjects (*n* = 362)	OR (95% CI), *p-*value
rs1800797/rs1818879			
H1: 11 GG	108.42 (24.1)	137.79 (19.0)	1.34 (1.01–1.79), 0.03
H2: 12 GA	326.58 (72.6)	547.21 (75.6)	0.84 (0.64–1.11), 0.23
H3: 21 AG	12.58 (2.8)	34.21 (4.7)	0.57 (0.30–1.11), 0.09
H4: 22 AA	2.42 (0.5)	4.79 (0.7)	ND
rs1800796/rs1818879			
H1: 11 GG	108.49 (24.1)	153.69 (21.2)	1.18 (0.89–1.56), 0.23
H2: 12 GA	159.51 (35.4)	246.31 (0.34)	1.07 (0.83–1.37), 0.59
H3: 21 CG	12.51 (2.8)	18.31 (2.5)	ND
H4: 22 CA	169.49 (37.7)	305.69 (42.2)	0.82 (0.64–1.05), 0.12
rs1800797/rs1800796/rs1818879			
H1: 111 GGG	96.97 (21.5)	123.5 (17.1)	1.34 (0.99–1.80), 0.05
H2: 112 GGA	159.58 (35.5)	244.80 (33.8)	1.07 (0.84–1.38), 0.55
H3: 121 GCG	11.32 (2.5)	15.38 (2.1)	ND
H4: 122 GCA	167.14 (37.1)	302.33 (41.8)	0.82 (0.64–1.04), 0.11
H5: 211 AGG	11.46 (2.5)	30.19 (4.2)	0.60 (0.30–1.19), 0.14
H6: 212 AGA	0 (0)	1.52 (0.2)	ND
H7: 221 ACG	1.26 (0.3)	2.94 (0.4)	ND
H8: 222 ACA	2.28 (0.5)	3.36 (0.5)	ND

CI, confidence interval; H, haplotype; ND, not determined; OR, odds ratio; SNPs, single-nucleotide polymorphisms. The SNPs are listed in the order: IL-6 rs1800797_G>A, rs1800796_G>C, and rs1818879_G>A.

The OR and 95% CI, and *p*-values were obtained by SHESIS test for haplotypes.

*p*-value < 0.05 was con.

LD analysis revealed a high level of linkage in patients with RA (**Supplementary Figure S1A**) between the rs1800797-rs1818879 and rs1800796-rs1818879 polymorphisms, while these SNPs were not linked in control subjects (**Supplementary Figure S1B**); this suggests a potential disease-causing allele linked to a specific haplotype in the patients with RA from our population. Individually, the SNPs did not correlate with increased serum IL-6 levels (**Supplementary Figures S2A–C**).

### 3.3 Association between circulating levels of IL-6 and clinical parameters in RA

In order to explore the relationship of IL-6 concentrations on the clinical and serological parameters of patients with RA, we reclassify the cohort on individual with low, middle, or high levels of IL-6 according to the IL-6 tertiles found in our cohort. The patients with RA with high levels of IL-6 (≥27.3 pg/mL) showed a longer morning stiffness time (*p* = 0.003), a higher functional disability score (*p* = 0.009), greater clinical activity (*p* < 0.001), and an increased number of tender and swelling joints (all *p* < 0.001); furthermore, they had higher levels of ESR (*p* = 0.006), hsCRP (*p* < 0.001), and RF (*p* = 0.033) (**Table 4**).

**Table 4:** Clinical, serological, and therapy characteristics according to IL-6 tertile.

Variables	Low (*n* = 72)	Middle (*n* = 78)	High (*n* = 75)	*p-*value
Demographic data				
Age, years, median (P_5_–P_95_)^a^	43 (24–69)	48.5 (23–70)	43 (24–73)	0.582
Clinical assessment				
Duration of illness, years, median (P_5_–P_95_)^a^	4 (1–17)	7 (1.5–32)	7 (1–22)	0.031
Morning stiffness, min, median (P_5_–P_95_)^a^	5 (0–120)	5 (0–180)	30 (0–360)	0.003
HAQ-DI, score, median (P_5_–P_95_)^a^	0.11 (0–1.5)	0.2 (0–1.7)	0.35 (0–1.5)	0.009
VAS, score, median (P_5_–P_95_)^a^	20 (0–80)	20 (0–80)	30 (0–100)	0.064
TJC, median (P_5_–P_95_)^a^	0 (0–24)	0 (0–20)	2 (0–24)	0.0004
SJC, median (P_5_–P_95_)^a^	0 (0–12	0 (0–20)	2 (0–24)	<0.0001
DAS28-ESR, score, median (P_5_–P_95_)^a^	2.72 (1.68–5.82)	3.07 (1.89–6.72)	3.88 (2.03–7.45)	<0.0001
SvH, score, median (P_5_–P_95_)^a^	2 (1–4)	2 (1–4)	2 (1–4)	0.90
Serological and inflammatory markers				
ESR, mmh, median (P_5_–P_95_)^a^	25 (9–47)	30 (7–55)	37 (7–56)	0.006
hsCRP, mg/L, median (P_5_–P_95_)^a^	4.75 (0–13.4)	6.34 (0–27.7)	19.6 (0–145.4)	<0.001
Rheumatoid factor, IU/mL, median (P_5_–P_95_)^a^	64.5 (8.6–566.9)	180 (10–650.6)	165.7 (8.6–942.5)	0.033
Anti-CCPs, U/mL, median (P_5_–P_95_)^a^	31.4 (0–421.2)	65.8 (0–520)	67.5 (0–431.1)	0.190
Current therapy scheme				
Monotherapy DMARDs, *n* (%)^b^	37 (51.39)	38 (48.72)	29 (38.67)	0.260
Bitherapy or more DMARDs, *n* (%)^b^	35 (48.61)	40 (51.28)	46 (61.33)	
Therapy scheme combined				0.760
DMARDs with or without NSAIDs, *n* (%)^b^	50 (69.44)	51 (65.38)	53 (70.67)	
DMARDs with GCs *n* (%)^b^	22 (30.56)	27 (34.62)	22 (29.33)	

Anti-CCPs, anti-cyclic citrullinated peptide antibodies; Cs, corticosteroids; DAS28, disease activity score 28; DMARDs, disease-modifying antirheumatic drugs; ESR, erythrocyte sedimentation rate; HAQ-DI, health assessment questionnaire disability index; hsCRP, high-sensitivity C-reactive protein; NSAIDs, nonsteroidal anti-inflammatory drugs; SJC, swollen joint count; SvH, Sharp van der Heijde Score; TJC, tender joint count; VAS, Visual Analog Scale.

Categories of IL-6 were assessed as follows: Low: IL-6 <10.6 pg/mL; Medium: IL-6 = 10.6–27.29 pg/mL; High: IL-6 ≥27.3 pg/mL.

a Data are expressed as the median using percentiles 5th–95th and Kruskal–Wallis test.

b Data are expressed as the *n* (%) using chi-square test. *p*-value <0.05 was considered statistically significant.

**Figure 1:**
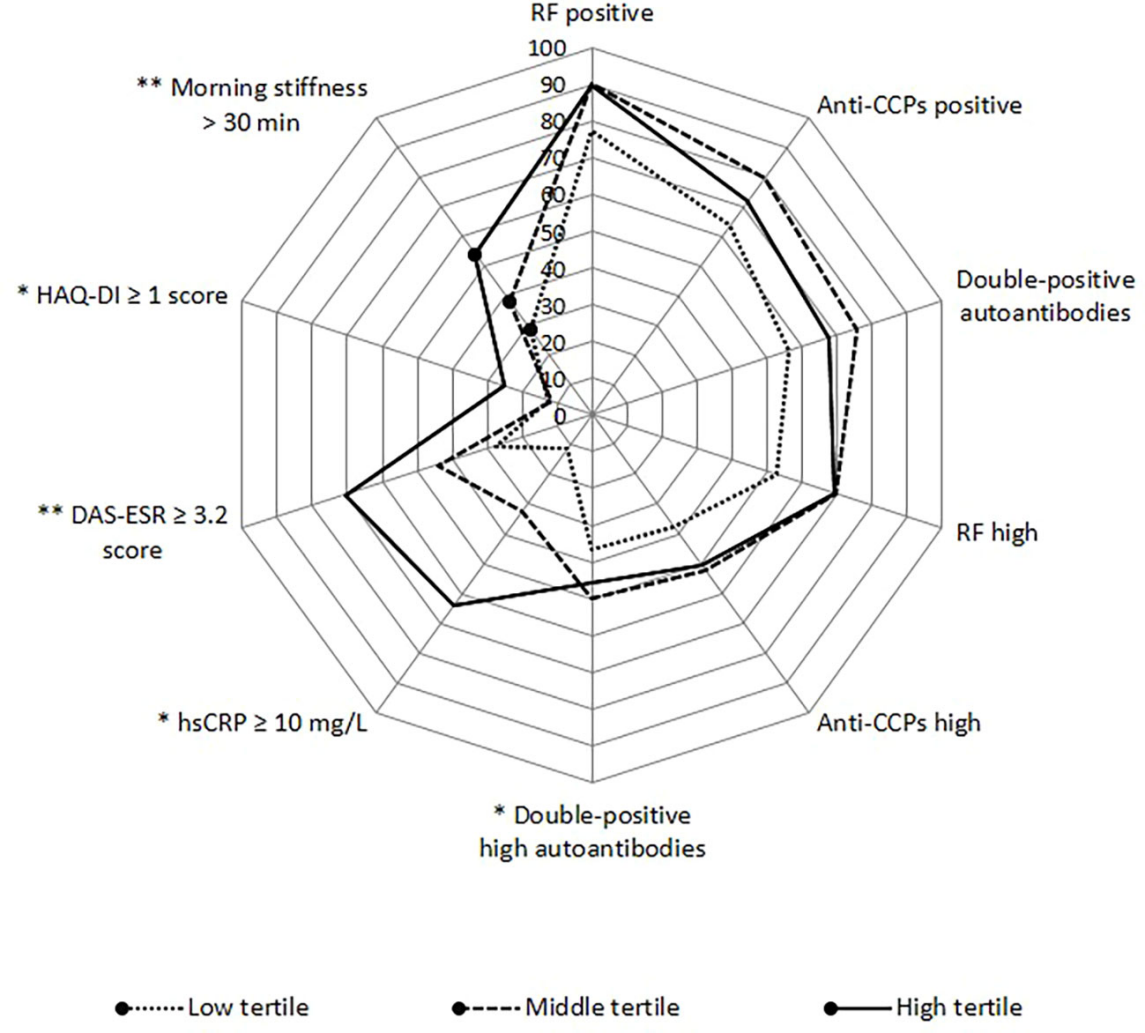
Proportion of serological and clinical alterations according to IL-6 tertiles. Anti-CCPs, anti-cyclic citrullinated peptide antibodies; DAS28, disease activity score 28; ESR, erythrocyte sedimentation rate; HAQ-DI, health assessment questionnaire disability index; hsCRP, high-sensitivity C-reactive protein; RF, rheumatoid factor. Categories: RF positive >20 IU/mL; Anti-CCPs positive >5 IU/mL; RF high >60 IU/mL; Anti-CCPs high >30 IU/mL; double-positive autoantibodies when levels were RF >20 IU/mL and anti-CCPs >5 IU/mL; double-positive high autoantibodies when levels were RF >60 IU/mL and anti-CCPs >30 IU/mL. Low tertile: IL-6 <10.6 pg/mL; medium tertile: IL-6 = 10.6–27.29 pg/mL; high tertile: IL-6 ≥27.3 pg/mL. *p-value < 0.05, **p-value < 0.01.

In addition, we analyzed the proportion of patients with RA with clinical and serological parameters predictive of severity of the disease, in relation to IL-6 tertile values, finding a significantly higher proportion of patients in the high tertile of IL-6 with morning stiffness >30 min (*p* < 0.01), functional disability defined by HAQ-DI ≥1 (*p* < 0.05), moderate–high clinical activity (*p* < 0.01), hsCRP ≥10 mg/L (*p* < 0.05), and double-positive (RF+/Anti-CCPs+) high autoantibodies (*p* < 0.05) (**Figure 1**).

### 3.4 *IL-6* haplotype and circulating levels of IL-6 in relation to severity parameters in RA

**Figure 2:**
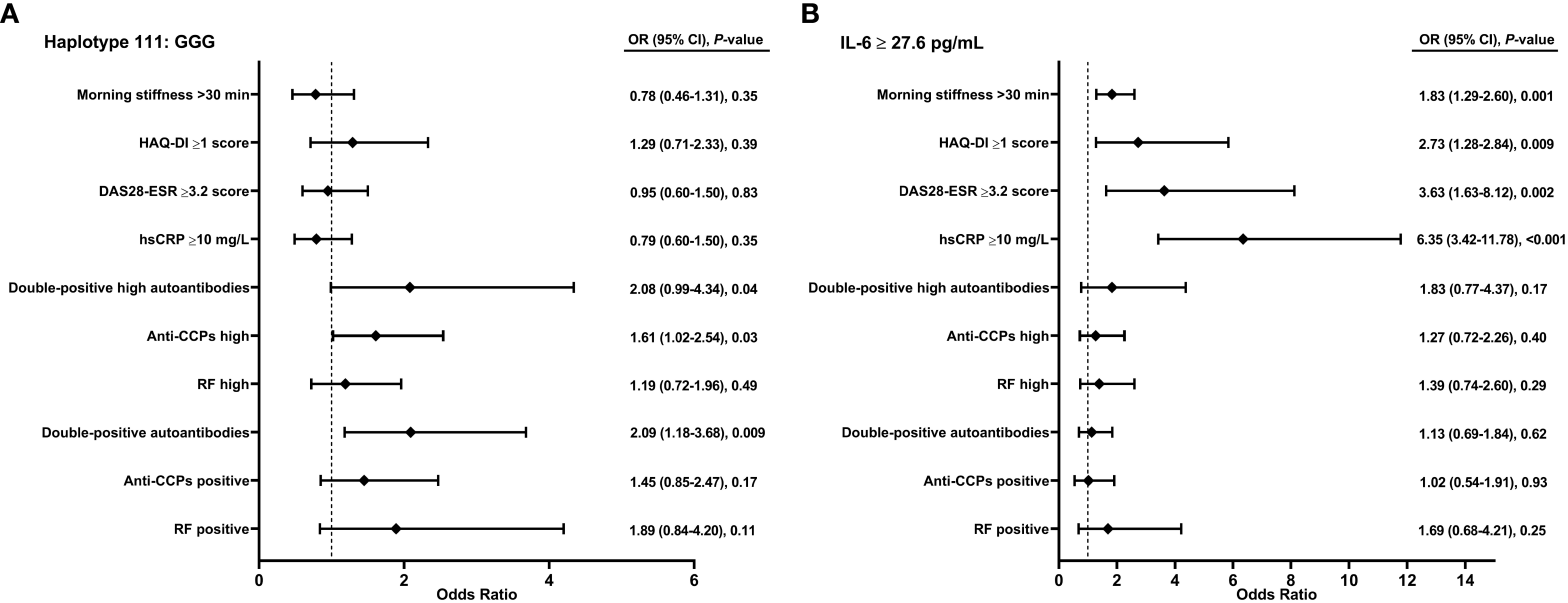
Association of haplotype and circulating levels of IL-6 on severity in patients with RA. **(A)** Haplotype. **(B)** Circulating levels of IL-6. Anti-CCPs, anti-cyclic citrullinated peptide antibodies; DAS28, disease activity score 28; ESR, erythrocyte sedimentation rate; HAQ-DI, health assessment questionnaire disability index; hsCRP, high-sensitivity C-reactive protein; RA, rheumatoid arthritis; RF, rheumatoid factor. Categories: RF positive >20 IU/mL; Anti-CCPs positive >5 U/mL; RF high >60 IU/mL; Anti-CCPs high >30 U/mL; double-positive autoantibodies when levels were RF >20 IU/mL and anti-CCPs >5 U/mL; double-positive high autoantibodies when levels were RF >60 IU/mL and anti-CCPs >30 U/mL. Figure **(A)** Odds ratio of univariate model. Figure **(B)** Odds ratio of multivariate model adjusted by age, sex, and therapy scheme combined. p-value < 0.05 was considered significant.

We explore the ORs associated with the severity parameters or poor prognosis of RA in individuals with the GG and GGG haplotypes with the highest circulating levels. The GG haplotype (rs1800797/rs1818879) was not associated with severity parameters or poor prognosis of RA and serum levels of IL-6. However, the GGG haplotype of the *IL-6* gene shows significant association with serologic status for double-positive autoantibodies, including RF+/anti-CCPs+ (OR = 2.09, 95% CI, 1.18–3.68, *p* = 0.009). It is also associated with high anti-CCPs+ levels (OR = 1.61, 95% CI, 1.02–2.54, *p* = 0.03) and with the double-positive high autoantibodies’ serologic status (OR = 2.08, 95% CI, 0.99–4.36, *p* = 0.04) (**Figure 2A**).

In an adjusted multivariate analysis accounting for age, sex, and therapy scheme, high circulating levels of IL-6 (≥27.3 pg/mL) are associated with increased susceptibility to morning stiffness ≥30 min (OR = 1.83, 95% CI, 1.29–2.60, *p* = 0.001), HAQ-DI ≥1 (OR = 2.73, 95% CI, 1.28–2.84, *p* = 0.009), DAS28-ESR ≥3.2 (OR = 3.63, 95% CI, 1.63–8.12, *p* = 0.002), and hsCRP ≥10 mg/L (OR = 6.36, 95% CI, 3.42–11.78, *p* < 0.001) (**Figure 2B**).

## 4 Discussion

We analyzed the role of three IL-6 SNPs and haplotypes on the susceptibility to develop RA and on the main clinical features on individuals diagnosed with RA. We found that haplotype GGG is associated with serological status to autoantibodies; furthermore, it suggests a role in susceptibility to RA, while the circulating levels of IL-6 are linked to features related to severity and poor prognosis in patients with RA.

Previous studies have reported that the SNPs rs1800797 (13) and rs1800796 (13–16) in the promoter region of the *IL-6* gene are associated with susceptibility to RA. However, the association of rs1800796 SNP with RA has not been established in populations from Turkey (23), Egypt (24), and Mexico (Mexican mestizo population) (25).

In a meta-analysis involving populations from Asia, the Middle East, Eastern Europe, Western Europe, and Latin America (15), the rs1800797 SNP was not linked to increased risk of developing RA. Similarly, in this study, individual SNPs did not show association with RA susceptibility, but the rs1800797 and rs1818879 SNPs, as well as the rs1800796 and rs1818879 SNPs, were found to be in strong LD. Likewise, other populations have reported high association between the SNPs rs1800797 and rs1818879 (26). The connection between these SNPs, despite their distant locations, could be explained by the formation of a loop that facilitates interaction between the promoter region (loci of the SNPs rs1800797 and rs1800796) and the 3′UTR region (rs1818879) of the IL-6 gene (27, 28).

In Turkish patients with RA, the GG haplotype (rs1800795 and rs2069837) (20) and the haplotype GCCGCT (rs1800797, rs1800796, rs1800795, rs1524107, rs2069840, and rs1474347) in Chinese patients with RA have been reported to be strongly associated with RA susceptibility (13). In the present study, the GG haplotype (rs1800797 and rs1818879) was associated, while the GGG haplotype (rs1800797, rs1800796, and rs1818879) showed a marginal association. It is important to consider that the Mexican population has a mestizo background; thus, ethnicity could potentially be a factor related to the variability in RA development. Some studies suggest that the Amerindian component in the Mexican population may play a role in susceptibility to RA (29).

The GGG haplotype was significantly associated with double-positive autoantibodies’ status (RF+/Anti-CCPs+) and double-positive high autoantibodies’ serologic status. A possible explanation of these findings is that SNPs could influence the expression of IL-6 (1, 10). IL-6 plays a key role as a regulator of both T-cell migration and activation, as well as B lymphocytes’ maturation into long-lived plasma cells, which is crucial in producing autoantibodies such as RF and anti-CCP (30). Furthermore, it has been shown that fibroblast-like synoviocytes (FLS) express CD40L and IL-6, while other studies suggest that the mechanism of FLS-dependent B-cell activation is subject to the involvement of both CD40L and IL-6, indicating that this cytokine participates in autoantibody production in RA (31, 32). Previous studies have demonstrated that patients with RA who are double-positive for autoantibodies (RF and anti-CCPs) have significantly higher IL-6 levels compared to seronegative patients or those positive for only a single autoantibody (33).

In the present study, circulating levels of IL-6 were not associated with individual SNPs or haplotypes, possibly because SNP rs1818879 does not participate directly in IL-6 synthesis although it has an indirect influence on IL-6 levels by binding to other SNPs located in the gene promoter region. Furthermore, it is important to consider that there are post-transcriptional and post-translational mechanisms that could be regulating the levels of the cytokine. For example, in the complementary chain of the IL-6 gene, the IL-6-AS1 gene is transcribed, the product of which is a long non-coding RNA that acts as an endogenous competitor to regulate IL-6 expression (28). On the other hand, soluble glycoprotein 130 (sgp130) acts as an antagonist of the trans-signaling mechanism by binding to the IL-6:sIL6R complex (34). Additionally, given that all patients in this study received anti-rheumatic therapy, we raise the possibility that treatment could mask the relationship between SNPs and circulating levels of IL-6.

Patients with RA treated with sulfasalazine experience a significant decrease in IL-6 levels, and this reduction in IL-6 is linked to improvements in clinical and laboratory measures of disease activity. This suggests that sulfasalazine may partly exert its disease-modifying effect by suppressing cytokine production (35). Additionally, in patients with RA with newly diagnosed disease and after 3 months of methotrexate treatment, radiological damage progression was correlated with IL-6 levels (36). In RA treatment, IL-6 blockade has become a first-line option, showing significantly greater clinical efficacy in patients who do not respond to conventional antirheumatic therapy or even to less conventional treatments. This indicated that IL-6-targeted drugs may reduce the burden of immune-inflammatory disease in a larger proportion of patients (37). However, in this study, monotherapy or combination therapies of antirheumatic drugs were not associated with circulating levels of IL-6.

According to circulating levels of IL-6 in this study, the median reported was similar to that reported in other studies on RA populations (38, 39), while others reported higher levels of circulating levels of IL-6 in patients with RA (8, 9, 40, 41). Several studies have found an association between IL-6 levels with clinical, serological, inflammatory, and biochemical markers in patients with RA (8, 9, 38–40). In the same way, we found a relationship between circulating levels of IL-6 and severity-related characteristics in patients with RA, such as morning stiffness, functional disability, moderate clinical activity, and higher levels of hsCRP. Symptoms like morning stiffness, pain, and functional disability could be linked to a peak in serum cortisol during the early morning hours in patients with RA who maintain elevated IL-6 levels (42). IL-6 acts on FLS, promoting their growth, increasing their survival, and modulating the synthesis of chemokines and matrix metalloproteinases (MMPs) (43); it also described that IL-6 can influence the differentiation and survival of osteoclasts, which express matrix metalloproteinase 3 (MMP-3); thus, osteoclasts induced by IL-6 may be involved in the joint destruction of RA (44). This partially explains the observed relationship with morning stiffness >30 min and HAQ-DI scores of 1 or higher, which may be linked to joint damage. IL-6 levels have been proposed as a marker of progression in bone and joint destruction during early RA stages (45). It has also been shown that progressive joint destruction tissue damage and persistent synovial inflammation lead to functional disability (46). IL-6 is involved not only in bone metabolism but also in immune response, hematopoiesis, and inflammation, and has been correlated with RA’s clinical activity and severity (47). As seen in our study, a DAS28-ESR score of 3.2 or higher was associated with high circulating levels of IL-6, similar to findings in other studies within the RA population (8, 9, 38–40).

Finally, in this study, elevated hsCRP levels ≥10 mg/L were associated with a high tertile of IL-6. Similarly, Boyapati et al. (48) reported that US patients with RA in the high tertile of IL-6 (64.7 pg/mL) had elevated CRP levels, moderately higher clinical disease activity at baseline, and greater joint damage. Furthermore, Zeb et al. (9) reported that, in Pakistani patients with RA, higher IL-6 levels correlated with CRP and disease severity. IL-6 directly promotes CRP synthesis through the binding of IL-6 to its receptor (IL-6R) on the hepatocyte membrane, activating the JAK-STAT pathway (2, 5). Furthermore, the exacerbated production of CRP in synovial tissue by FLS could be through the mechanism associated with CD32/64-p38 activation and NF-kB signaling (49), which further aggravates the inflammatory process in RA.

Other mechanisms related to IL-6 regulation could be related to the trans-presentation process, where circulating IL-6 binds to the membrane receptor of dendritic cells (IL-6Rα) and subsequently the IL-6/IL-6Rα complex binds to gp130 expressed on CD4^+^ T cells and forms a gp130 homodimer, activating signaling JAK. This interaction is necessary for the generation of Th17 cells (2). Furthermore, a computational model revealed that IL-6 and IL-17 (interleukin-17) signaling promotes the formation of the STAT3–NF-kB complex, followed by its binding to the promoter regions of NF-kB target genes to accelerate inflammatory responses, including IL-6 production (50).

### 4.1 Strengths and limitations

This study demonstrates the association of a new haplotype with serological status in RA. However, the results found should be interpreted with caution, as this study only included patients from southern Mexico. Furthermore, using other methods such as eQLT identification from different tissues and cell types, as well as implementing an *in silico* analysis, would allow us to better understand how the *IL-6* gene and genetic variants influence susceptibility to RA or circulating levels of IL-6, as well as the molecular mechanisms of IL-6 related to inflammation and immune regulation.

## 5 Conclusion

In conclusion, the GGG haplotype of the *IL-6* gene is linked to double-positive high autoantibodies, while elevated circulating levels of IL-6 are connected to clinical severity features in Mexican patients with RA. Additional studies are necessary to clarify the mechanism behind the SNPs.

## Data Availability

The raw data supporting the conclusions of this article will be made available by the authors, without undue reservation.
