# Supplementary material for: Interleukin-6 levels and their haplotypes are associated with serological autoantibodies status and clinical activity in rheumatoid arthritis
Source: Front Immunol. 2025 Nov 6;16:1679644. doi: 10.3389/fimmu.2025.1679644 (PMC12631633; doi:10.3389/fimmu.2025.1679644)
Supplement: Supplementary file 1 [file Table1.docx]

Supplementary Material

# Supplementary Figures

| (A) 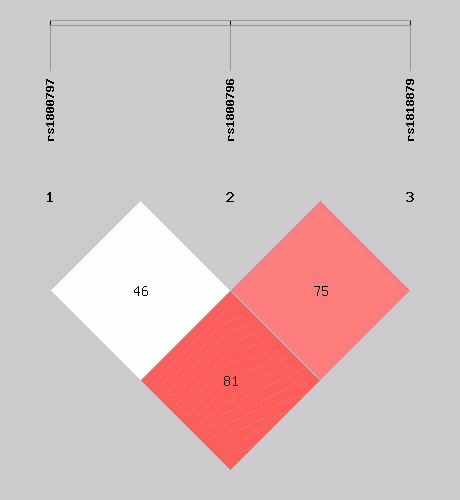 | (B) 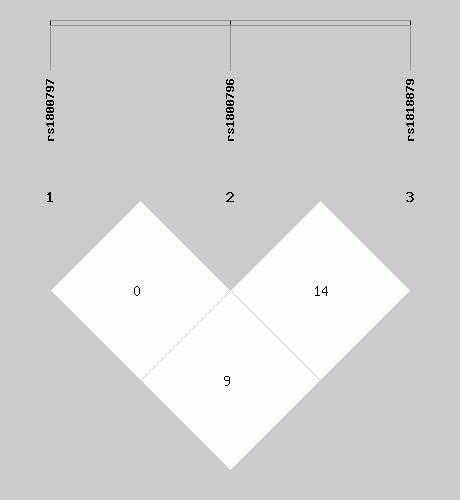 |
| --- | --- |
| **Supplementary Figure 1. Linkage disequilibrium (LD) test of *IL-6* gene SNPs in RA patients (A) and control subjects (B).** Haplotype frequencies and LD were calculated using SHEsis software. Red area represents higher levels of LD. A D´ value of 100 indicates a complete LD between two markers and a D´ value of 0 indicates complete linkage equilibrium. rs1800796-rs1800797 (D' = 0.463, r^2^ = 0.008); rs1800796-rs1818879 (D' = 0.757, r^2^ = 0.144); rs1800797-rs1818879 (D' = 0.818, r^2^ = 0.095). | |

| (A)   | (B)   | (C)   |
| --- | --- | --- |
| **Supplementary Figure 2. Serum levels of IL-6 according to genotypes for SNPs (A) rs1800797, (B) rs1800796, and (B) rs1818879 in RA patients.** Differences between genotypes, using the Mann-Whitney U test (p5-p95 th). *P* value <0.05 was considered significant according to the genotype of comparison. | | |
